# Correlation between Processing Parameters, Morphology, and Properties of Injection-Molded Polylactid Acid (PLA) Specimens at Different Length Scales

**DOI:** 10.3390/polym15030721

**Published:** 2023-01-31

**Authors:** Laura Meinig, Regine Boldt, Yvonne Spoerer, Ines Kuehnert, Markus Stommel

**Affiliations:** 1 Leibniz Institute of Polymer Research Dresden (IPF), Hohe Str. 6, 01069 Dresden, Germany; 2 Institute of Material Science, Technical University Dresden, 01062 Dresden, Germany

**Keywords:** PLA, injection molding, inhomogeneous morphology, micromechanical tests

## Abstract

Polylactic acid (PLA) is one of the most promising bioplastic representatives that finds application in many different areas, e.g., as single-use products in the packaging industry, in the form of mulch film for agriculture, or in medical devices. For the development of new areas, especially in terms of long-term applications and the production of recyclable products, the material properties controlled by processing must be known. The state of the art is investigations at the global scale (integral values) without consideration of local structure inhomogeneities and their influence on the material properties. In this work, morphological, thermal, and mechanical properties of injection-molded PLA tensile bars are investigated at different length scales (global and local) as a function of processing parameters. In addition to the processing parameters, such as melt temperature, mold temperature, and cooling time in the mold, the influence of the D-isomer content on the crystallization behavior and the resulting material properties are investigated. The material was found to form crystalline structures only when cooled in a mold tempered above T_g_. In addition, PLA with a lower content of D-isomer was found to have a higher degree of crystallinity. Since the mechanical properties obtained by tensile tests could not be correlated with the degree of crystallinity, detailed analysis were performed showing a characteristic inhomogeneous morphology within the tensile bars. By means of micromechanical investigations on samples with different microstructure ranges, the relationship between local morphology and failure behavior could be explained.

## 1. Introduction

The environmental awareness is increasing and with it the desire to avoid petro-based plastics as far as possible or to use more sustainable alternatives. Especially in the area of disposable products such as food packaging, mulch films in agriculture, or plastic bags, the desire for a more environmentally friendly alternative is great [1]. Polylactide (PLA) represents one of these promising alternatives [2]. PLA is a bio-based and biodegradable bioplastic that can be polymerized from renewable resources such as corn, sugarcane, or sugar beets via the intermediate stage of polylactic acid [1,3].

Typical applications for PLA include short-life products such as packaging or biomedical applications [4,5,6]. In the future, the fields of application will also be extended more and more to long-term applications, such as in the automotive or electrical industry [2,3]. Currently, thermoplastics such as polyamide (PA) and polyoxymethylene (POM) are used for these technically demanding applications [7]. Both semi-crystalline thermoplastics exhibit good heat resistance and impact strength as well as good sliding and wear properties because of their high crystallinity. PLA is as well a semi-crystalline thermoplastic material, but in comparison to PA and POM, it crystallizes very slowly. Due to the rather slow crystallization behavior of PLA, injection-molded components are largely amorphous. Since the cycle times are kept as low as possible, the material does not have the required time to crystallize sufficiently [4,8,9,10]. It is known that for the adaptation or modification of the application-specific properties, the material has to be crystalline [2,11,12].

In order to improve the crystallization behavior, nucleating agents are usually added [10]. Alternatively, amorphous molded specimens can be subjected to a subsequent heat treatment known as annealing. In this process, the specimens are heated to a temperature above the glass transition temperature to stimulate the polymer chains, which are frozen in the molten state, to post-crystallize [2]. Due to the increased temperature (>T_g_), however, deformation of the components and thus the dimensional stability cannot be guaranteed [13]. Another possibility to increase the crystallinity is to keep the material in the mold above T_g_ for a longer cooling time [14,15]. Consequently, the dimensional accuracy of the components is ensured. In addition, the degree of crystallization is increased by shear, which forces the polymer chains to be oriented, resulting in increased nucleation density. The shear experienced by the material during injection molding can be controlled, for example, by the set melt temperature.

Furthermore, in case of PLA, the crystallization behavior depends not only on temperature and time but also on stereochemistry and molecular weight [14]. PLA can be prepared from two optically active forms, L- and D-lactic acid. The lactic acid molecules differ only in the position of the OH group at the central carbon atom. Different forms of PLA (PDLLA, PLLA, and PDLA) can then be synthesized from the molecules of lactic acid. The crystallinity, glass transition behavior, and mechanical properties of the synthesized PLAs are significantly influenced by the ratio of enantiomeric lactic acid molecules [14,16].

This shows that the crystallinity of PLA is influenced by processing parameters such as mold temperature, melt temperature, and cooling time as well as the stereochemistry of the macromolecules. As a result of the changed crystallinity, the morphology and mechanical properties change.

With the aim to optimize the processing parameters in such a way that the crystallization behavior of the PLA can be improved without addition of additives, this work is divided into three parts:

(i) The influence of mold temperature and cooling time on the degree of crystallinity and, in consequence, on the mechanical properties was investigated;
(ii) Based on the resulting optimized processing parameters, the influence of melt temperature on crystallization kinetics and the material properties was analyzed;
(iii) Investigations of local morphological and mechanical properties were performed.

The crystallization behavior and final crystallinity were investigated using DSC, polarized optical microscopy, X-rays, and AFM. The mechanical properties were determined by means of tensile tests in the macro and micro range. The investigations of the material properties are carried out both globally on the entire specimen and locally in specific areas of the specimens. The detailed local investigations of the injection-molded test specimens are intended to provide information about the microstructure and its influence on the morphological, thermal, and mechanical behavior.

## 2. Materials and Methods

### 2.1. Materials

Two biodegradable polylactid grades, i.e., PLA 3100HP (MFR: 24 g/10 min at 210 °C and 2.16 kg [17], T_melt_ = 173 °C, 0.5% D-lactide content) and 3001D (MFR: 22 g/10 min at 210 °C and 2.16 kg [18], T_melt_ = 168 °C, 1.5% D-lactide content), were purchased from Nature Works LLC., Plymouth, MN, USA. The materials were supplied in pellet form. The molar masses determined by SEC are similar for both materials.

### 2.2. Processing

Before injection molding, the materials were dried for 8 h at 80 °C to remove any moisture. In order to investigate the influence of different processing parameters on the morphological, thermal, and mechanical properties, 1BA tensile bars were fabricated with melt temperatures of 180 °C, 190 °C, 195 °C, 200 °C, and 210 °C. The mold temperatures were set at 25 °C, 50 °C, and 100 °C, and the cooling time in the mold was adjusted at 15 s, 30 s, 60 s, and 150 s. The sample labeling used letters indicating the PLA grade (HP = PLA 3100HP and D = PLA 3001D) and numbers representing the processing parameters (T_melt_-T_mold_-t_cool_) (Table 1).

### 2.3. Preparation

**Figure 1:**
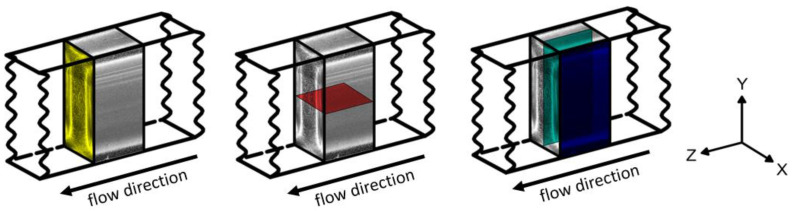
Specimen sawn out of the tensile bar with the respective preparation planes: XY (yellow), XZ (red, middle (M)), and YZ (blue, skin (S); turquoise, core (C)).

Pieces with a length of approx. 1 cm were sawn out of the center of the injection-molded tensile bars. Thin sections were prepared from the sawn-out specimen using a Leica RM 2265 rotary microtome (Wetzlar, Germany). For the DSC measurements, thin sections were made from the center and across the cross-section of the injection-molded tension bars. The preparation layers are shown in Figure 1. In order to also analyze the influence of flow direction, thin sections were prepared both parallel and perpendicular to the flow direction. In order to be able to distinguish the thin sections, an additional identification letter was added to the specimen nomenclature. The letters M, S, and C stand in the same order for the position of the section in the middle, at the skin, and in the core. For AFM measurements, an ultramicrotome EM FC7 from Leica (Wetzlar, Germany) was used to prepare a flat surface area of 100 × 100 µm perpendicular to the flow direction.

### 2.4. Characterization

Optical microscopy was used to study the morphology within the injection-molded PLA parts, as well as the thermal and micromechanical properties. The investigations of the injection-molded specimens were performed with an Axio Imager.A1m light microscope from Zeiss (Oberkochen, Germany) equipped with an Axiocam 305 microscope camera and Zen Core software v2.7 from Zeiss. The microscopy images were acquired in polarization contrast and transmitted light and brightfield mode. The same microscope setup and a device built at IPF-Dresden were used for the micromechanical studies [19].

The hot-stage experiments were performed at a Linkam Imaging station (Waterfield, Tadworth, UK) equipped with a Linkam hot stage (Waterfield, Tadworth, UK). Images during hot-stage experiments were captured using a FLIR Systems microscope camera (Wilsonville, OR, USA) in polarization contrast and transmitted light and brightfield mode and processed using Linkam’s LINK software v1.2.17 (Waterfield, Tadworth, UK). For the hot-stage studies, thin sections of the starting pellets of PLA 3100HP and PLA 3001D were heated to 180 °C and 210 °C, respectively; held there for 2 min; and then cooled to 100 °C. The samples were held at this temperature for 10 min and then cooled to room temperature at 50 K/min and used for measurements. The isothermal holding temperature of 100 °C corresponds to the mold temperature during injection molding.

Atomic force microscopy studies were performed using a Dimension FastScan AFM instrument (Bruker-Nano, Billerica, MA, USA). A SCANASYST-FLUID+ silicon nitride sensor (Bruker, USA) with a tip radius of 2 nm and a nominal spring constant of 0.7 N/m served as the cantilever. The nominal value was 0.02 V.

WAXS studies were performed at the MiNaXS beamline of the German Electron Synchrotron (Hamburg, Germany). The beam size was 20 μm × 30 μm, and the X-ray wavelength was 0.1048 nm. The exposure time was 0.5 s. The images of the 2D patterns were acquired with a Lambda 9M detector. For the evaluation of the degree of crystallization, the 2D patterns were integrated azimuthally from 55–110°. Thin sections were scanned in 0.05 mm increments from the skin towards the center. The data were processed and analyzed using dpdak software (version 1.5.0) [20]. The results were plotted in a one-dimensional diffractogram. The intensity was normalized between 0 and 1 and plotted over a diffraction angle of 2Θ.

The molar mass and polydispersity were determined by means of size exclusion chromatography (SEC) according to the PS-2A standard by dissolving an average of 2 mg of PLA in chloroform. The solution was pumped through a 1PL MIXED-B-LS separation column at a flow rate of 1.0 mL/min. Detection of the molecules was performed using an RI detector.

The melting and crystallization behavior was analyzed by Differential Scanning Calorimetry (DSC) using a DSC Q1000 (TA Instruments, Waters, MA, USA). The crystalline fraction of the samples was calculated using the following equation [21]:

(1) $$X_{C}=\frac{{\Delta H}_{m}-{\Delta H}_{c}}{{\Delta H}_{m}^{0}}\times 100\%$$

ΔH_m_ indicates the melting enthalpy, which results from the integration of the endothermic melting peak. ΔH_c_ indicates the sum of the integrals of the exothermic crystallization effects such as cold crystallization and recrystallization. ΔH^0^_m_ is the enthalpy of melting of a 100% crystalline PLA material, which is given in the literature as 93.7 J/g [21]. The measurements were taken in a heat/cool/heat mode in a temperature range from—80 °C to 220 °C with a ramp rate of 10 K/min. For characterization of the process-related properties, the first heating cycle was used to determine the thermal effects, e.g., enthalpy of fusion, glass transition temperature (T_g_), and melting temperature (T_melt_). The thin sections used for the thermal studies had an average mass of 0.6 mg.

The 1BA tensile bars were stretched with a testing machine from Zwick/Roell (Ulm, Germany) according to DIN EN ISO 527 at room temperature and a testing speed of 50 mm/min. The data were evaluated as average values of five tested samples each.

The micromechanical investigations were performed on 20 µm thin sections taken parallel to the flow direction. The test speed was 0.05 mm/s, and the force transducer used was designed for a maximum load of 2 N. The tests were performed at room temperature. The clamping length was set at 5 mm. The data were evaluated using the average values of four tested samples.

## 3. Results

### 3.1. Effect of Mold Temperature and Cooling Time on Global Material Properties

To determine the influence of mold temperature and cooling time on material properties, the specimens were processed under variation of the mold temperature (25 °C, 50 °C, and 100 °C) and cooling time (15 s, 30 s, 60 s, or 150 s). At a mold temperature of 100 °C, the cooling time for the PLA 3001D had to be adjusted and extended to 60 s since the samples were difficult to remove from the mold at a cooling time of 30 s. The melt temperature was kept constant at 210 °C.

**Figure 2:**
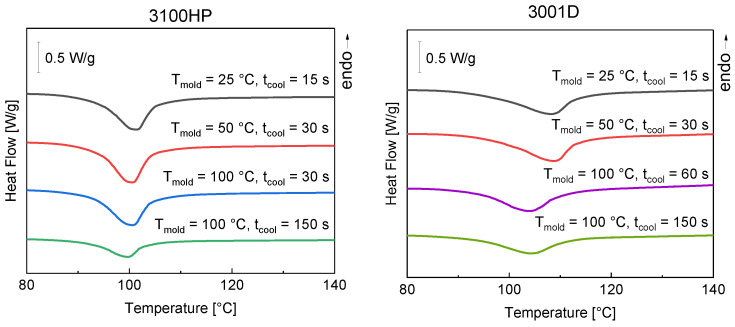
DSC curves of the first heating cycle of injection-molded tensile bars made of PLA 3100HP (left) and PLA 3001D (right) produced with a melt temperature of 210 °C and cooled in a temperature-controlled mold at 25 °C, 50 °C, and 100 °C for 15 s, 30 s, 60 s, and 150 s.

First, the influence of mold temperature and cooling time on crystallization behavior was investigated. The cold crystallization peaks determined from the first heating cycle are presented in Figure 2.

For all samples, an exothermic cold crystallization peak at around 100 °C was determined. This means that the material is not completely crystallized. A significant reduction of the cold crystallization enthalpy could only be achieved by increasing the cooling time (Table 2).

Based on the DSC data, the degrees of crystallization of the injection-molded tensile bars were determined using Equation (1). The results are shown in Table 2. It can be seen that the degree of crystallinity is almost constant for both PLA materials when the cooling time is 60 s or less. Even when the mold temperature increases, the cooling time is too short to affect the amount of crystalline fraction. If, on the other hand, the cooling time in the mold is extended, a significant increase in the crystalline fraction can be seen. It becomes clear that at a sufficiently high mold temperature T_mold_ = 100 °C > T_g_, the material requires a certain time (>60 s) to produce a significant increase in the crystalline fraction. This is more evident in PLA 3100HP than in 3001D, which is due to the different D-lactide content.

The different states of crystallinity were also verified by polarized light microscopy (Figure S1). Crystalline structures were detected only in the samples processed at a mold temperature of 100 °C and a cooling time of 150 s.

**Figure 3:**
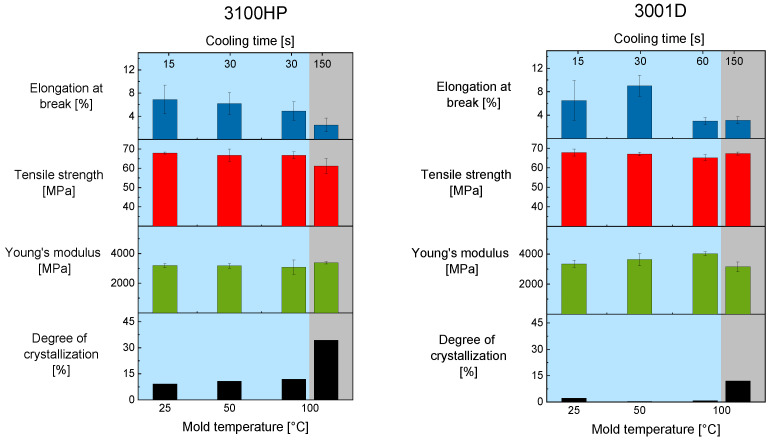
Mechanical properties such as Young’s modulus (green), tensile strength (red), and elongation at break (blue) as well as the degree of crystallization (black) of the injection-molded tensile bars of PLA 3100HP (left) and PLA 3001D (right) processed with T_melt_ 210 °C and T_mold_ = 25 °C, 50 °C, and 100 °C.

To determine the effect of process-induced degree of crystallinity on the mechanical properties tensile, tests were performed. The results are shown in Figure 3. The tests show that there are no major differences in Young’s modulus and tensile strength for PLA 3100HP processed with a cooling time of 30 s or less. This was to be expected with the degree of crystallinity. By extending the cooling time in a mold tempered at 100 °C, a trend is seen. Even though the deviations are very large, the tensile strength decreases, while the Young’s modulus increases. A similar trend is observed for the elongation at break. With increasing cooling time and thus with increasing degree of crystallinity, the elongation at break decreases. It is noteworthy that even very small differences in the degree of crystallinity affect the elongation at break significantly.

This was not observed for the samples made of PLA 3001D. The elongation at break does not correlate with the degree of crystallinity. Furthermore, in contrast to the samples made of PLA 3100HP, PLA 3001D shows an increased Young’s modulus for samples processed with a cooling time of 60 s or less even when the degree of crystallinity is constant. The test specimens of PLA 3001D show a decreased Young’s modulus when the degree of crystallinity increases.

It was expected that a higher degree of crystallization leads to an increased Young’s modulus [11,12]. This could not be observed and is not yet explained. For this reason, further investigations were performed (iii).

### 3.2. Effect of Melt Temperature on the Material Properties

The influence of melt temperature on the crystallization behavior of PLA was first investigated by means of hot-stage experiments. The experiments were performed at 180 °C and 210 °C, as they were intended to be the minium and maximum processing temperature of the melt.

**Figure 4:**
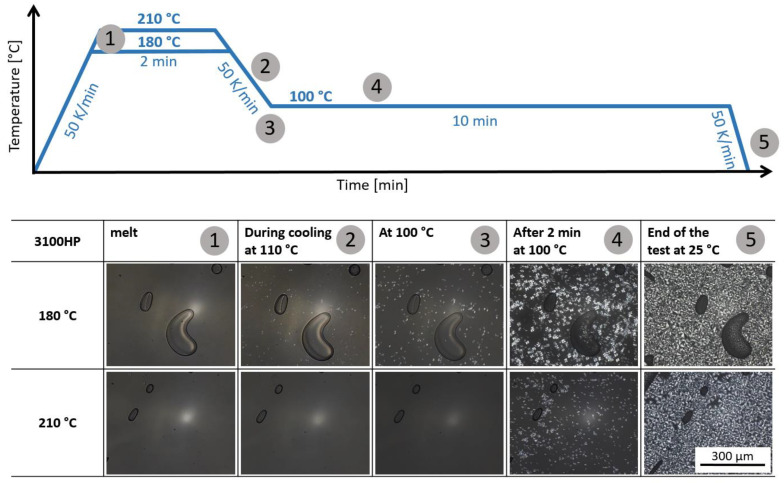
Light microscopic images of the material 3100HP during the hot-stage experiments in the molten state at 180 °C and 210 °C (1), during cooling to 100 °C (2), at the beginning of isothermal crystallization at 100 °C (3), after 2 min at 100 °C (4), and at the end of the test at room temperature (5).

Figure 4 shows the images from the hot-stage experiments of 3100HP at different states. It is seen that PLA 3100HP is in a fully molten state at 180 °C and 210 °C. When cooling from 180 °C, the first crystalline structures are already visible at about 110 °C. Comparing the samples at the beginning of the isothermal cooling time at 100 °C, it is noticeable that crystalline structures are only visible in the sample molten at 180 °C. The sample molten at 210 °C shows no crystalline structures at the same time. Only after a cooling time of a few seconds at 100 °C could the the formation and growth of crystalline structures be observed. At the end of the experiments, crystalline structures with spherulites of approx. 40–50 μm size are visible in both samples over the entire image section.

**Figure 5:**
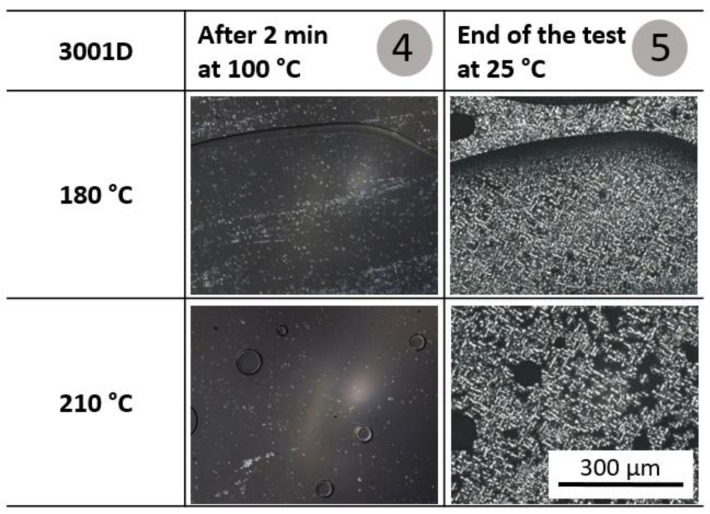
Light microscopic images of the material 3001D during the hot-stage experiments starting from 180 °C and 210 °C after 2 min at 100 °C (4) and at the end of the test at room temperature (5).

In comparison to PLA 3100HP, PLA 3001D crystallizes significantly more slowly. The images from the hot-stage experiments of PLA 3001D are shown in Figure 5 (full overview, Figure S2). Only after a cooling time of about 60 s at 100 °C could the first crystalline structures could observed. At the end of the experiments, the spherulites have a diameter of about 20–30 μm. In the sample molten at 210 °C, larger amorphous regions are visible between the spherulites.

In summary, it can be concluded from the hot-stage experiments that both PLA grades crystallize at 100 °C regardless of the initial melting temperature. Only the onset of crystallization is influenced by the melting temperature. Cooling from a 180 °C melt results in an earlier onset of crystallization than cooling from 210 °C. This could be attributed to incompletely molten crystal structure fragments acting as the material’s own nuclei. Since the incompletely molten structures are very small, they could not be imaged with the maximum achievable magnification.

**Figure 6:**
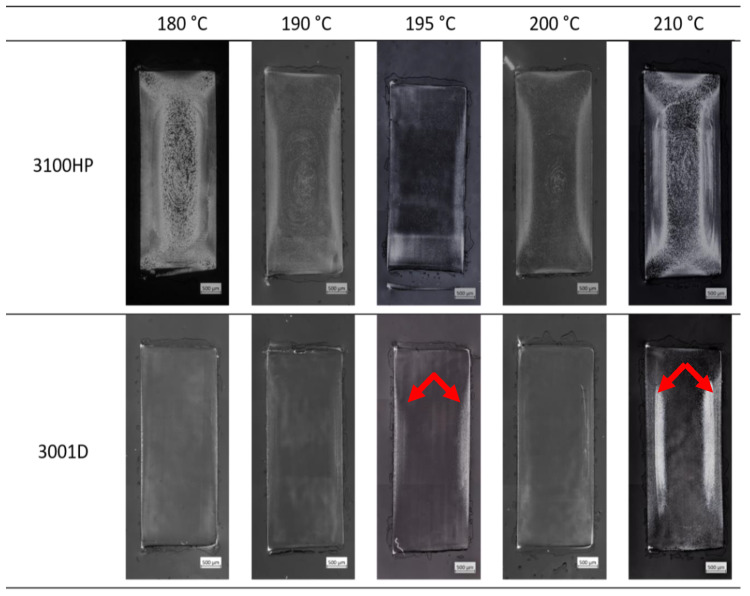
Polarized light microscope images of sample cross-sections of PLA 3100HP (top row) and 3001D (bottom row) processed by injection molding with different melt temperatures but constant mold temperature of 100 °C and constant cooling time of 150 s.

For the injection-molding process, this means that both 180 °C and 210 °C could be suitable parameters for melt processing to obtain crystalline tensile bars. Consequently, both PLA 3100HP and 3001D were injection-molded at these temperatures. In addition, both PLA grades were injection-molded at melt temperatures of 190 °C, 195 °C, and 200 °C. Following the results in Section 3.1, the mold temperature was set to 100 °C and the cooling time was set to 150 s. After injection molding, cross-sections were prepared from the middle of the tensile bars for optical microscopy analysis and DSC. Figure 6 shows the polarized optical light microscopic images. As expected from the results of the previous hot-stage experiments, many small crystalline structures are seen in the cross-sections of the specimens processed at 180 °C and 210 °C. Since individual spherulites are observed in the center of the cross-sections, it appears at first glance that the crystalline structures are mainly located in the center. Only at second glance do the very small crystalline structures in the skin areas become visible. In contrast, the samples processed at 190 °C, 195 °C, and 200 °C show only small, crystallized regions, mainly located at the skin. In the case of PLA 3001D, crystalline areas are only seen at the skin and only in samples processed at a melt temperature of 195 °C and 210 °C, respectively. Comparing the microscopic images of PLA 3001D and 3100HP, it can be seen that PLA 3001D always has a smaller amount of crystalline structures. Since the processing parameters were the same, the reason for the lower percentages of crystalline structures must be due to the higher D-isomer content of PLA 3001D compared to PLA 3100HP. In addition, there is always an inhomogeneous morphology. The reason for the intrinsically different morphology and its influence on mechanical properties are explained in more detail in the following section (iii).

**Figure 7:**
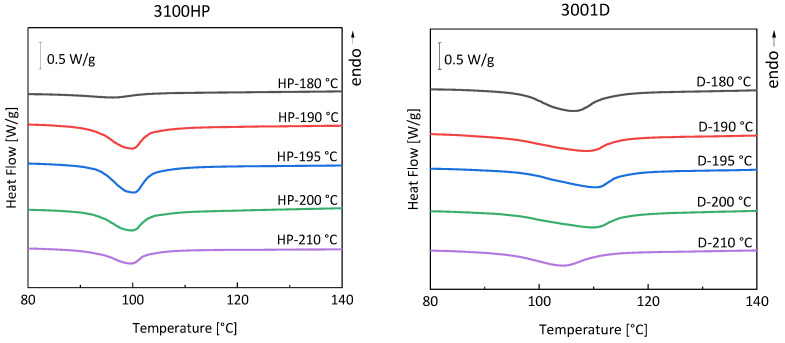
DSC curves of the first heating cycle of injection-molded tensile bars made of PLA 3100HP and PLA 3001D produced with melt temperatures of 180 °C, 190 °C, 195 °C, 200 °C, and 210 °C and cooled in a temperature-controlled mold at 100 °C for 150 s.

The influence of melt temperature on thermal properties was analyzed by DSC. The DSC curves of the cold crystallizations are shown in Figure 7. The melting effects are shown in Figure S3 in the supplementary material. For PLA 3100HP, the cold crystallization enthalpies are minimal at 180 °C and 210 °C. In contrast, a maximum enthalpy is observed at 195 °C. This means that the samples processed at melt temperatures of 180 °C and 210 °C do not have as much material post-crystallized as the sample processed at 195 °C. For the sample processed at 180 °C, it is also evident that the cold crystallization temperature is 96 °C, which is about 4 K lower than the other samples, which post-crystallize at about 100 °C. Since the melt temperature of 180 °C is only just above the temperature at which no endothermic melting effect is observed, it is possible that incompletely molten nuclei or crystal fragments are still present in the melt. These initially solidify as they cool from the melt. If the material is subsequently reheated, nuclei are formed by aggregation of polymeric segments. This behavior is also described in the literature [22]. Since these domains already have a certain size, further chains can attach more quickly.

The samples of PLA 3001D show a strong shift in cold crystallization temperatures. The cold crystallization temperature first increases from 106.2 °C and drops to a minimum of 104.4 °C at a melt temperature of 210 °C.

**Figure 8:**
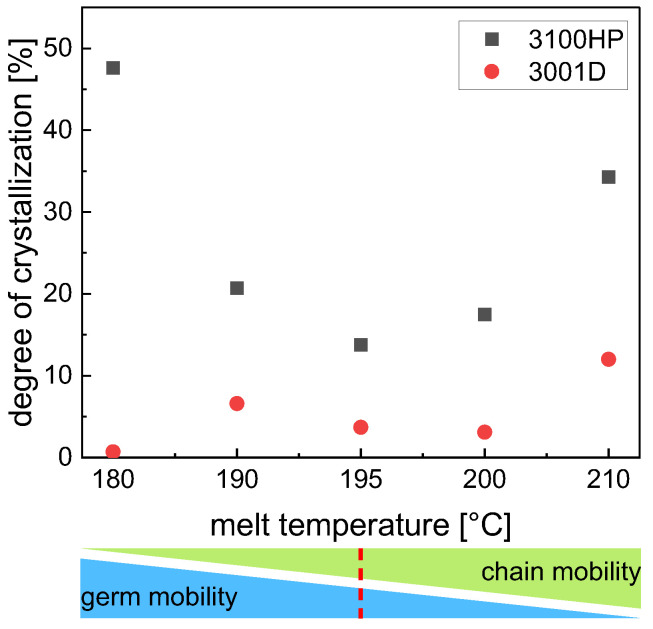
Degree of crystallization obtained from DSC first heating scans as a function of melt temperature during processing.

The degrees of crystallization determined from the DSC data (Equation (1)) are shown in Figure 8. Compared to PLA 3100HP, PLA 3001D always shows lower degrees of crystallization at the same processing procedure because of a different D-isomer content.

PLA 3100HP processed at a melt temperature of 180 °C has a maximum degree of crystallization of 48%. According to the literature [23], the reason for the high degree of crystallization may be due to nucleation by the material’s own incompletely molten nuclei. Due to the low chain mobility at melt temperatures of 180 °C (just above the melting temperature), stable nuclei or crystal fragments remain in the melt and serve as nucleation surfaces. The degree of crystallization of PLA 3100HP decreases with increasing melt temperature, shows a minimum at 195 °C, and increases again with increasing melt temperature.

**Figure 9:**
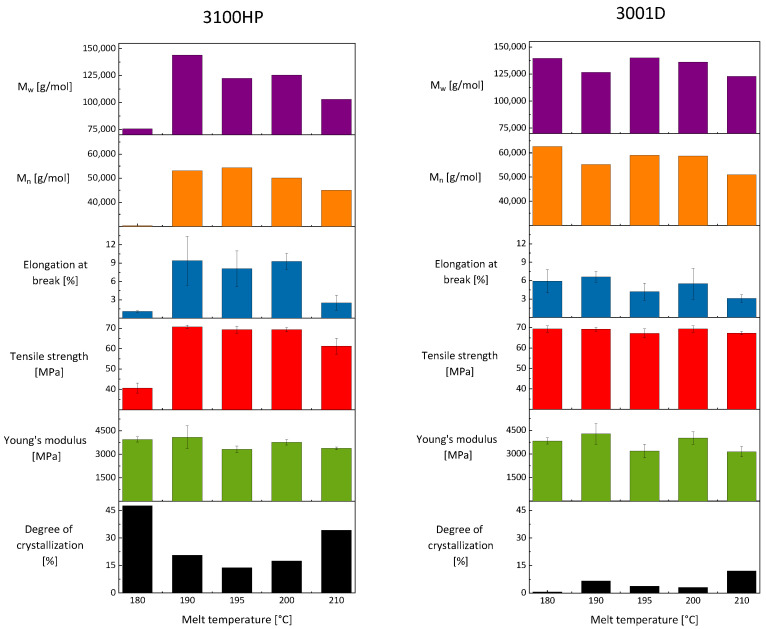
Mechanical properties such as Young’s modulus (green), tensile strength (red), and elongation at break (blue) as well as the degree of crystallization (black) and the number (orange) and mass-averaged (purple) molecular masses as a function of melt temperature of injection-molded tension rods made of PLA 3100HP (left) and PLA 3001D (right) processed at melt temperatures of 180 °C, 190 °C, 195 °C, 200 °C, and 210 °C and cooled for 150 s in a mold tempered to 100 °C.

The minimum of degree of crystallization at 195 °C indicates an overlap of two different effects, namely the small amount of material intrinsic nuclei and reduced chain mobility. Both result in minimized crystallization kinetics. We assume that on the one hand, the temperature of 195 °C is too high for the existence of intrinsic nuclei of PLA. On the other hand, the temperature of 195 °C is too low for sufficient chain mobility, which is necessary for crystal growth. In addition to temperature, the chain mobility and thus the degree of crystallinity is also affected by the molar masses. When the molar masses are significantly reduced, an increasing degree of crystallization is observed (Figure 9). The molar mass reduction is due to process-related thermal-mechanical stresses [12]. The resulting shortened polymer chains promote parallel formation and thus crystallization of the material.

To investigate the mechanical properties, tensile tests were carried out (Figure 9). Samples of 3100HP processed at melt temperatures of 180 °C and 210 °C have the lowest elongation at break and the lowest tensile strength. The Young’s modulus shows the lowest values at 195 °C and 210 °C. A good combination of mechanical properties such as high elongation at break, high tensile strength, and high modulus of elasticity was measured for the specimens injection-molded at melt temperatures of 190 °C and 200 °C, respectively. The elongation at break here is about 9% at both temperatures. The tensile strength shows values of about 70 MPa, and the Young’s modulus is in the range of 4000 MPa.

In contrast to PLA 3100HP, the lowest mechanical properties of 3001D are evaluated for the samples processed at melt temperatures of 195 °C and 210 °C. The combination of high Young’s moduli, tensile strengths, and elongations at break was achieved when the specimens were processed at melt temperatures of 180 °C and 190 °C, respectively. In this case, elongations at break of 6–7% were obtained as well as tensile strengths of 67 MPa and Young’s modulus values of 3800 MPa and 4300 MPa.

It becomes clear that the mechanical properties cannot be correlated with the processed-induced morphological and thermal properties. It has to be noticed that the mechanical properties are integral values determined for the entire tensile bars. Considering the morphology we observed in the cross-sections of the specimens (Figure 6), it was expected that it would not be possible to explain different mechanical properties on a global scale. Therefore, detailed investigations on the local scale were necessary (iii).

### 3.3. Local Properties of PLA Tensile Bars

**Figure 10:**
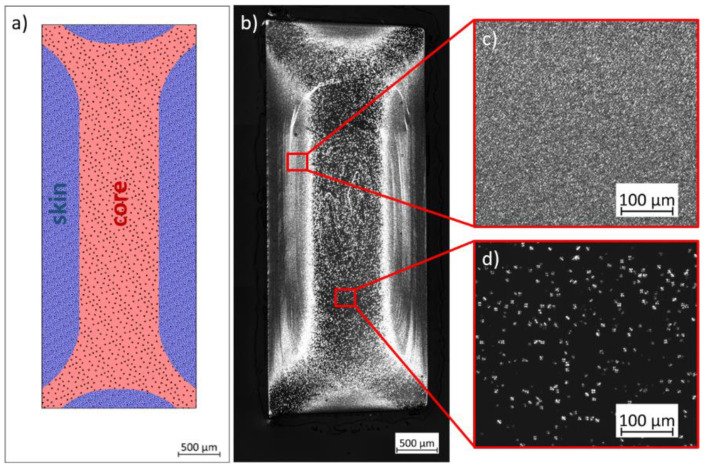
(**a**) Schematic representation of the skin and core areas created by injection molding, (**b**) exemplarily shown at sample HP-210-100-150 with the corresponding detailed images from the (**c**) skin and (**d**) core area.

The cross-sections of the injection-molded specimens from Figure 6 not only show morphological differences in comparison to each other but also an inhomogeneous structure distribution within the cross-section. The reason for the inhomogeneous microstructure distribution can be attributed to processing. A temperature gradient is formed in the material due to the rapid cooling of the melt when it hits the tempered mold wall. At the skin, the heat of the melt can be dissipated directly through the mold, resulting in the skin layer solidifying first. The core area of the specimen cools more slowly than the skin layer due to the poor thermal conductivity of the material. This causes the material to flow at different rates in the skin and core regions. The different velocities of the melt flow cause high shear of the material in the skin and transition areas between the skin and core layers. The strong orientation in the skin and layer close to the skin leads to a high nucleation density and thus to many small crystalline structures, as it can be seen in Figure 10c. Due to the lower shear in the core region, significantly fewer but larger crystalline structures are visible (Figure 10d). The skin and core structures each account for about 50% of the total cross-sectional area. The distribution of the skin and core layers is shown by sample HP-210-100-150 in Figure 10b and as a simplified model of the skin and core regions in Figure 10a.

**Figure 11:**
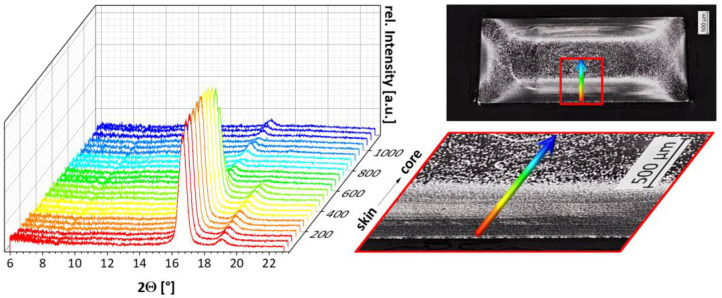
X-ray diffractograms (left) taken over the cross-section of sample HP-210-100-150 which was processed with a melt temperature of 210 °C, a mold temperature of 100 °C and a cooling time in the mold of 150 s.

These observations were confirmed by WAXS studies of the sample HP-210-100-150. Here, the measurement is made across the cross-section to the center at 50 µm intervals, as it is seen in Figure 11b. The positional X-ray diffractograms are shown in Figure 11a. They show the diffraction-angle-dependent intensities across the cross-section. It can be seen that the reflection intensities decrease sharply from the skin towards the core. The intensity is high if the X-ray beam was diffracted at many crystals and low if the diffraction was only at a few crystals. This suggests a high proportion of crystalline structures in the skin layer and few crystalline structures in the core region. This correlates well with the light microscopic investigations, in which a greater amount of crystalline structures was observed in the skin region than in the core.

**Figure 12:**
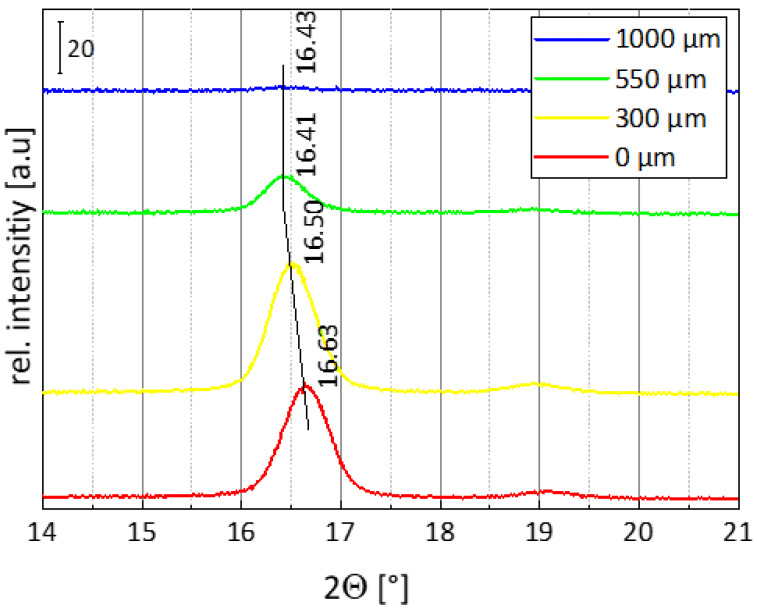
Four exemplary diffractograms measured at different sample positions across the cross-section showing the characteristic diffraction angles of the α and α’-modification of PLA.

From the diffraction angles, information about crystal modification was obtained. The peak positions are shown on the example of four diffractograms from the skin, transition, and core area in Figure 12. It is seen that the angles at 16.63° are directly located at the skin and 16.50° at a sample position of 300 µm. The diffraction reflections in the transition area occur at 16.41°, and directly in the center of the sample, reflections are found at a diffraction angle of 16.43°.

According to the literature, the determined angles are characteristic for the α and α’-modification of PLA. Diffraction angles of 16.7° are characteristic for the thermodynamically stable α-modification. The α’-modification, on the other hand, is observed at diffraction angles of 16.4° [24,25]. Moreover the α’-modification shows a slightly larger lattice dimension than the α-modification [24,26]. This indicates that in the skin, mainly the α-modification is formed, while in the core, the α’-modification predominates.

**Figure 13:**
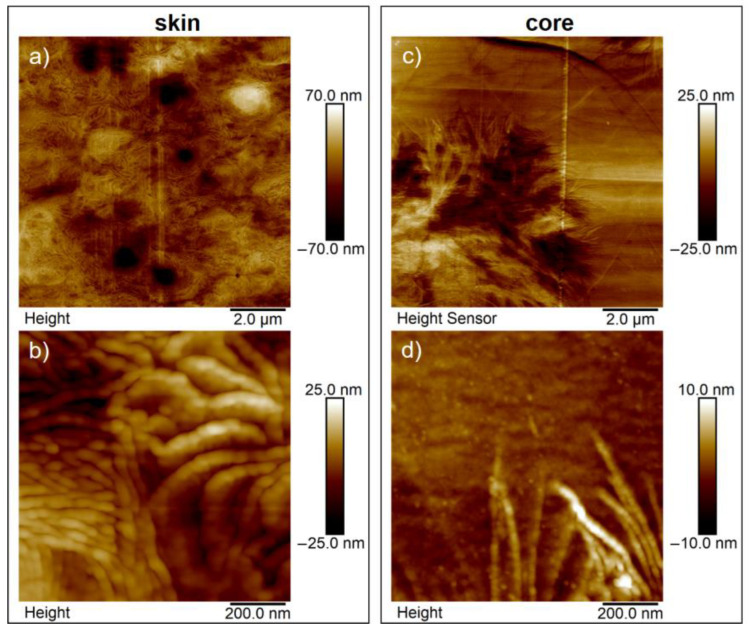
AFM images of the skin (**a**,**b**) and core structure (**c**,**d**) of sample HP-210-100-150 taken over the cross-section perpendicular to the flow direction.

In addition to WAXS measurements, AFM measurements were performed on sample HP-210-100-150 to investigate the lamellar structure and the size of the crystalline structures in the skin and core area. AFM measurements were made in areas of the skin and core perpendicular to the direction of flow. In the skin region (Figure 13a), a homogeneous distribution of many small crystalline structures is seen. The core region (Figure 13c) is characterized by a few large spherulites embedded in an amorphous matrix. It can also be seen that the lamellae in the core region grew into the amorphous matrix. At higher resolution, the lamellae are clearly visible. In the skin region (Figure 13b), lamellae with a thickness of approx. 60 nm were measured, while the lamella thickness in the core region (Figure 13d) was about 30 nm. The measured lamella thicknesses are intended as a rough guide and cannot be determined unambiguously and comprehensively from the AFM images. The reason for this is the different orientation of the lamellae as well as a certain image blurring. According to the literature, the different lamella thicknesses are due to different crystallization temperatures [27,28]. Cho and Strobl [28] found in their studies that the lamella thickness increases with decreasing crystallization temperature. This is consistent with the results of our AFM studies.

The crystallization temperature also has an influence on which crystal modification is formed. The literature states that at temperatures < 100 °C, PLA crystallizes completely in the α’-modification. From 100–120 °C, α- and α’-modification crystallize at the same time. Above a temperature of 120 °C, PLA crystallizes only in the form of the α-modification [29,30].

However, from this point of view, in contrast to our results from WAXS, the α’-phase would have been expected in the skin region of the injection-molded samples due to the faster cooling to the mold temperature of 100 °C. In the core region, on the other hand, the α-phase would have been more expected due to the slower cooling. The different results may be due to the superposition of several effects during injection molding. When the melt is injected, the material is highly sheared close to the skin region, leading to a local temperature increase. This may cause degradation of the material, resulting in a molecular weight loss, which in turn results in changed flow and crystallization behavior.

**Figure 14:**
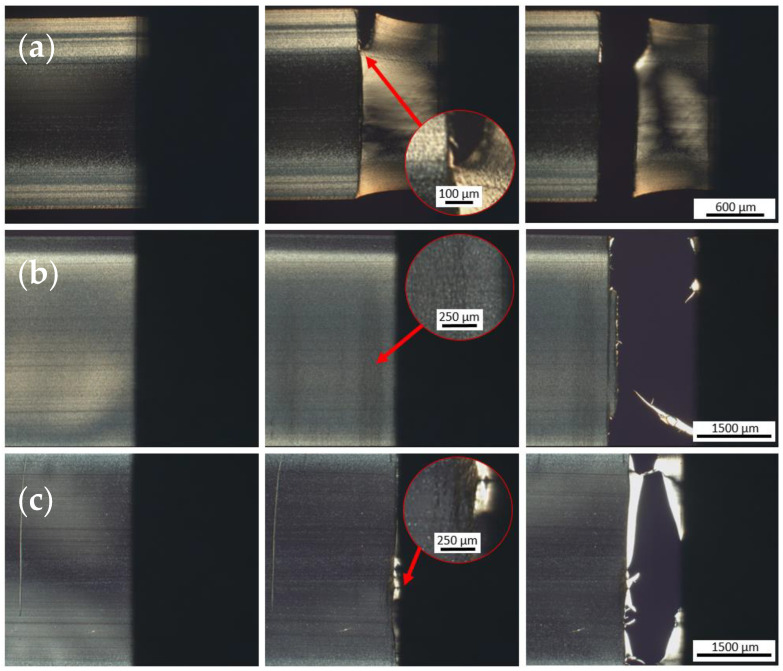
Snapshots during micromechanical testing of specimens (**a**) HP-210-100-150-M, (**b**) HP-210-100-150-S, and (**c**) HP-210-100-150-C, showing from left to right the conditions at the beginning of the measurement, at the beginning of the first failure, and at the end of the test after complete failure of the specimen.

To investigate the local mechanical properties, microtensile tests were carried out on thin sections in the direction of flow under room temperature. For this purpose, thin-section specimens were first taken over the entire specimen thickness (Figure 14a) so that both skin and core structures were included in the specimen.

**Figure 15:**
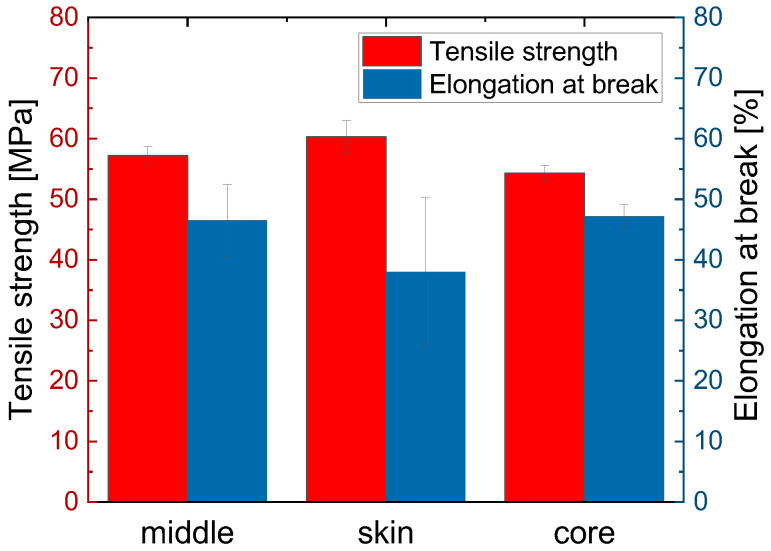
Characteristic values such as tensile strength and elongation at break determined for specimens HP-210-100-150-M, HP-210-100-150-S, and HP-210-100-150-C from the measured values of micromechanical tests.

The sequences of the microtensile tests are shown in Figure 14. When the HP-210-100-150-M specimen was stretched (Figure 14a), the elongation of the specimen became visible in the clamping region. Subsequent cracking of the specimen began in the area that WAXS measurements indicated as the highly crystalline skin region. Subsequently, the specimen failed in the form of an abrupt and smooth crack. The average tensile strength was 57.2 ± 1.5 MPa, and the average elongation at break was 46.5 ± 5.9%. The results of the mechanical tests are summarized in Figure 15. Both the thin section of sample HP-210-100-150 and the corresponding tensile bar, which was characterized in more detail in Section 3.2, can be considered as a kind of composite material consisting of crystalline and amorphous regions. The tensile strength of the tensile bar is 61.2 ± 3.9 MPa, which is slightly higher than that of the thin section. When comparing the tensile bar and the thin section, it should be noted that the measurements on the tensile bar and the thin sections were performed with different speeds, force sensors, and clamping devices.

The highly crystalline specimen HP-210-100-150-S, taken from the skin region according to Figure 14b, initially showed microfractures near the clamping region under tensile load, which formed perpendicular to the flow direction. Subsequently, the specimen failed, with a highly brittle nature directly in the clamping area. Under tensile loading, the specimen was able to reach a maximum tensile strength of 60.3 ± 2.7 MPa and an elongation at break of 38.0 ± 12.2%.

The less-crystalline specimen HP-210-100-150-C (Figure 14c) showed slight microcracking transverse to the flow direction at the beginning of the failure. For the tensile strength, a value of 54.3 ± 1.3 MPa was obtained. The average value of the tensile strengths of the skin and core samples, 57.3 MPa, corresponds exactly to the value of sample HP-210-100-150-M, which is composed of skin and core structures, thus confirming the assumption of a composite effect of the different structures. The elongation at break of the core sample was 47.2 ± 1.9%, which is greater than the elongation at break of the skin sample. The reason for this is the higher percentage of amorphous regions determined by WAXS and AFM studies. In addition, it can be clearly seen in the AFM images from Figure 13 that the spherulites grown in the skin region are directly adjacent to each other due to their growth density. As described in the literature, the strain and thus initially the stress have to be transferred via the narrow amorphous boundary regions between the spherulites or directly via the amorphous regions in the lamellae [31]. In contrast, in the core region, the lamellae of the isolated spherulites are not bounded by adjacent spherulites but grow unhindered into the amorphous matrix. It is conceivable that this ingrowth leads to good interspherulitic bonding and thus to better force transfer between the amorphous and crystalline phases. This is also described by Razavi and Wang [31], who investigated the mechanical properties of PLA below and above T_g_, noting that the mechanical behavior in the glassy state (below T_g_) can be described as a interplay between the chain network and a structure of intermolecular (between the molecules) bonding. They further described that the amorphous region in semi-crystalline polymers below T_g_ can occur in three places: (1) intra-spherulitic between the lamellae in the spherulites, (2) inter-spherulitic at the interfaces of adjacent spherulites, and (3) in regions where no spherulites have formed. Thereby, the amorphous regions at the spherulite interfaces play a crucial role in the force transmission of the tensile load. The connection of amorphous and crystalline regions, both within the lamellae and between the spherulites, is achieved by so-called bridge chains. These bridging chains are chain segments that are partly embedded in the crystalline domains and partly dangled out into the amorphous domain, where they form networks with other non-crystalline chains. Chain segments that dangles out of the crystalline region but do not form networks with other chains also occur but are not called bridging chains [31]. If a load is now applied to the material, the force is initially transmitted across the interfaces (2) between the amorphous and crystalline regions. The failure in these regions is due to the bridging chains being pulled out of the crystalline regions, as it requires less force than the mutual sliding of the chains in the disordered amorphous regions. Razavi and Wang [31] described that in partially crystallized PLLA, failure occurs mainly within the spherulites (1) or at the interfaces of the spherulites (2). The reason for this is probably the presence of a robust chain network in the amorphous regions between the spherulites [31]. In fully crystallized PLLA, only amorphous regions of forms (1) and (2) are present, so the force is predominantly dissipated through the interfaces between the spherulites.

In addition to the influence of the degree of crystallization, the crystal modification also affects the mechanical properties. Cocca et al. [32] found that material consisting of the α-modification has higher elastic moduli but also lower elongation than material consisting mainly of the α’-modification due to its ordered and densely packed crystal form. PLA composed of the α’-phase can achieve higher elongation due to conformational disorder. This is because the amorphous phase transfers the load to the crystals and absorbs the imposed strain [32]. This would explain both the higher tensile strengths of the thin sections from the skin region and the higher strains of the specimens from the core region.

## 4. Conclusions

In this work, it is shown with the help of morphological, thermal, and mechanical investigations that the injection-molding parameters such as mold temperature, melt temperature, and cooling time in the mold have a significant influence on the global and local properties of tensile specimens.

In order to obtain a crystalline component without the addition of additives or subsequent heat treatment, the mold temperature must be set to a temperature above T_g_. Otherwise, the injection-molded parts are completely amorphous.

Furthermore, the crystalline content is increased significantly by extending the cooling time in the mold. Compared to PLA 3100HP, PLA 3001D shows slower crystallization behavior under the same processing conditions, which is due to the higher proportion of D-isomers. The higher proportion of D-isomers has a hindering influence on the chain movement because of the mirrored molecular configuration.

In addition, PLA 3100HP shows a minimum of degree of crystallization at a melt temperature of 195 °C, which can be attributed to the change in molecular weights. It was observed that the degree of crystallinity does not correlate with the integral mechanical properties. Only by detailed investigations of the semi-crystalline tensile bars could the correlation between crystallinity and mechanical properties be explained. Within the tensile bars, different local morphologies were observed, which can be divided into skin and core regions. The skin region characterized by many small crystalline structures has a higher tensile strength but also a lower elongation at break than the less-crystallized core region.

Finally, it can be concluded that for the interpretation of the results on a global scale, the knowledge of properties on the local scale are necessarily required.

## Figures and Tables

**Table 1:** Injection-molding process parameters for the production of 1BA tension bars.

Sample	Melt Temperature (°C)	Mold Temperature (°C)	Cooling Time (Sec)
HP-210-25-15	210	25	15
HP-210-50-30	210	50	30
HP-210-100-30	210	100	30
HP-210-100-150	210	100	150
D-210-25-15	210	25	15
D-210-50-30	210	50	30
D-210-100-60	210	100	60
D-210-100-150	210	100	150
HP-180-100-150	180	100	150
HP-190-100-150	190	100	150
HP-195-100-150	195	100	150
HP-200-100-150	200	100	150
HP-210-100-150	210	100	150
D-180-100-150	180	100	150
D-190-100-150	190	100	150
D-195-100-150	195	100	150
D-200-100-150	200	100	150
D-210-100-150	210	100	150

**Table 2:** Non-isothermal DSC data of PLA 3100HP and PLA 3001D processed with a melt temperature of 210 °C under variation of mold temperature (T_mold_) and cooling time (t_c_).

		3100HP					3001D			
T_mold_ (°C)	t_c_ (sec)	T_c_ (°C)	ΔH_c_ (J/g)	ΔH_m_ (J/g)	Χ_c_ (%)	t_c_ (sec)	T_c_ (°C)	ΔH_c_ (J/g)	ΔH_m_ (J/g)	Χ_c_ (%)
25	15	101.4	39.6	48.2	9.1	15	108.4	37.4	39.5	2.2
50	30	100.6	42.0	52.0	10.8	30	108.7	39.0	39.3	0.3
100	30	100.5	39.7	50.9	12.0	60	104.0	37.3	38.1	0.8
100	150	99.6	21.0	53.0	34.3	150	104.4	29.5	40.7	12.0

## Data Availability

All data are available in the article or the Supplementary Material.

## Supplementary Materials

The following supporting information can be downloaded at: https://www.mdpi.com/article/10.3390/polym15030721/s1, Figure S1: Polarized light microscope images of sample cross-sections of PLA grades 3100HP (top row) and 3001D (bottom row) processed by injection molding with different mold temperatures and different cooling times of 15 s, 30 s, and 150 s but constant melt temperature of 210 °C; Figure S2: Light microscopic images of the material 3001D during the hot-stage experiments in the molten state, during cooling to 100 °C, at the beginning of isothermal crystallization at 100 °C, after 2 min at 100 °C, and at the end of the test at room temperature starting from a 180 °C (top row) and 210 °C (bottom row) hot melt; Figure S3: DSC curves of the first heating cycle of injection-molded tensile bars made of PLA 3100HP and PLA 3001D produced with melt temperatures of 180 °C, 190 °C, 195 °C, 200 °C, and 210 °C and cooled in a temperature-controlled mold at 100 °C for 150 s.

## References

[1] Ilyas R., Sapuan S. (2020). Biopolymers and biocomposites: Chemistry and technology. Curr. Anal. Chem..

[2] Di Lorenzo M.L., Androsch R. (2018). Industrial Applications of Poly(Lactic Acid).

[3] Tănase E.E., Râpă M., Popa O. Biopolymers based on renewable resources—A review. Proceedings of the International Conference Agriculture for Life, Life for Agriculture.

[4] Castro-Aguirre E., Iniguez-Franco F., Samsudin H., Fang X., Auras R. (2016). Poly(lactic acid)-Mass production, processing, industrial applications, and end of life. Adv. Drug Deliv. Rev..

[5] Murariu M., Dubois P. (2016). PLA composites: From production to properties. Adv. Drug Deliv. Rev..

[6] Lim L.T., Auras R., Rubino M. (2008). Processing technologies for poly(lactic acid). Prog. Polym. Sci..

[7] Brinkmann S., Schmachtenberg E. (2013). Saechtling Kunststoff Taschenbuch.

[8] Vadori R., Mohanty A., Misra M. (2013). The Effect of Mold Temperature on the Performance of Injection Molded Poly (Lactic Acid)-Based Bioplastic. Macromol. Mater. Eng..

[9] De Meo A., De Santis F., Pantani R. Effects of Rapid Cavity Temperature Variations on the Crystallinity of PLA. Proceedings of the 35th International Conference of the Polymer-Processing-Society (PPS).

[10] Tabi T., Ageyeva T., Kovacs J. (2022). The influence of nucleating agents, plasticizers, and molding conditions on the properties of injection molded PLA products. Mater. Today Commun..

[11] Södergård A., Stolt M. (2002). Properties of lactic acid based polymers and their correlation with composition. Prog. Polym. Sci..

[12] Farah S., Anderson D., Langer R. (2016). Physical and mechanical properties of PLA, and their functions in widespread applications—A comprehensive review. Adv. Drug Deliv. Rev..

[13] Kassos N., Kelly A.L., Gough T., Gill A. (2020). Acceleration of crystallisation rate in injection moulded PLLA by stereocomplex formation. Mater. Res. Express.

[14] Harris A.M., Lee E. (2008). Improving mechanical performance of injection molded PLA by controlling crystallinity. J. Appl. Polym. Sci..

[15] De Santis F., Volpe V., Pantani R. (2017). Effect of Molding Conditions on Crystalli zation Kinetics and Mechanical Properties of Poly(lactic acid). Polym. Eng. Sci..

[16] Endres H.-J., Siebert-Raths A. (2009). Technische Biopolymere: Rahmenbedingungen, Marktsitutation, Herstellung, Aufbau und Eigenschaften.

[17] NatureWorks (2022). Ingeo™ Biopolymer 3100HP Technical Data Sheet.

[18] NatureWorks (2022). Ingeo™ Biopolymer 3001D Technical Data Sheet.

[19] Fischer M., Pöhlmann P., Kühnert I. (2019). Morphology and mechanical properties of micro injection molded polyoxymethylene tensile rods. Polym. Test..

[20] Benecke G., Wagermaier W., Li C., Schwartzkopf M., Flucke G., Hoerth R., Zizak I., Burghammer M., Metwalli E., Müller-Buschbaum P. (2014). A customizable software for fast reduction and analysis of large X-ray scattering data sets: Applications of the new DPDAK package to small-angle X-ray scattering and grazing-incidence small-angle X-ray scattering. J. Appl. Crystallogr..

[21] Shen T., Ma P., Yu Q., Dong W., Chen M. (2016). The effect of thermal history on the fast crystallization of poly(L-lactide) with soluble-type nucleators and shear flow. Polymers.

[22] Sangroniz L., Cavallo D., Mu A. (2020). Self-nucleation effects on polymer crystallization. Macromolecules.

[23] Elias H. (2001). Makromoleküle Band 2: Physikalische Strukturen und Eigenschaften.

[24] Di Lorenzo M.L., Androsch R. (2019). Influence of α′-/α-crystal polymorphism on properties of poly (l-lactic acid). Polym. Int..

[25] Zhang J., Duan Y., Sato H., Tsuji H., Noda I., Yan S., Ozaki Y. (2005). Crystal modifications and thermal behavior of poly(L-lactic acid) revealed by infrared spectroscopy. Macromolecules.

[26] Kalish J.P., Zeng X., Yang X., Hsu S.L. (2011). A spectroscopic analysis of conformational distortion in the α′ phase of poly (lactic acid). Polymer.

[27] Lotz B. (2017). Crystal polymorphism and morphology of polylactides. Synthesis, Structure and Properties of Poly(Lactic Acid).

[28] Cho T.-Y., Strobl G. (2006). Temperature dependent variations in the lamellar structure of poly(l-lactide). Polymer.

[29] Zhang J., Tashiro K., Tsuji H., Domb A.J. (2008). Disorder-to-order phase transition and multiple melting behavior of poly(L-lactide) investigated by simultaneous measurements of WAXD and DSC. Macromolecules.

[30] Zhang J., Tashiro K., Domb A.J., Tsuji H. (2006). Confirmation of disorder α form of poly(L-lactic acid) by the X-ray fiber pattern and polarized IR/Raman spectra measured for uniaxially-oriented samples. Macromol. Symp..

[31] Razavi M., Wang S.-Q. (2019). Why is crystalline poly (lactic acid) brittle at room temperature?. Macromolecules.

[32] Cocca M., Di Lorenzo M.L., Malinconico M., Frezza V. (2011). Influence of crystal polymorphism on mechanical and barrier properties of poly(l-lactic acid). Eur. Polym. J..

