# Supplementary material for: Correlation between Processing Parameters, Morphology, and Properties of Injection-Molded Polylactid Acid (PLA) Specimens at Different Length Scales
Source: Polymers (Basel). 2023 Jan 31;15(3):721. doi: 10.3390/polym15030721 (PMC9921645; doi:10.3390/polym15030721)
Supplement: Supplementary file 1 [file polymers-15-00721-s001.zip › polymers-2172091-supplementary.pdf]

Supplementary data

Correlation between Processing Parameters, Morphology, and Properties of Injection-Molded Polylactid Acid (PLA) Specimens at Different Length Scales

Laura Meinig [1], Regine Boldt [1,*], Yvonne Spoerer [1], Ines Kuehnert [1], and Markus Stommel [1,2]

[1] Leibniz Institute of Polymer Research Dresden (IPF), Hohe Str. 6, 01069 Dresden, Germany; meinig@ipfdd.de (L.M.); spoerer@ipfdd.de (Y.S.); kuehnert@ipfdd.de (I.K.); stommel@ipfdd.de (M.S.)

[2] Institute of Material Science, Technical University Dresden, 01062 Dresden, Germany

* Correspondence: boldt@ipfdd.de (R.B.)

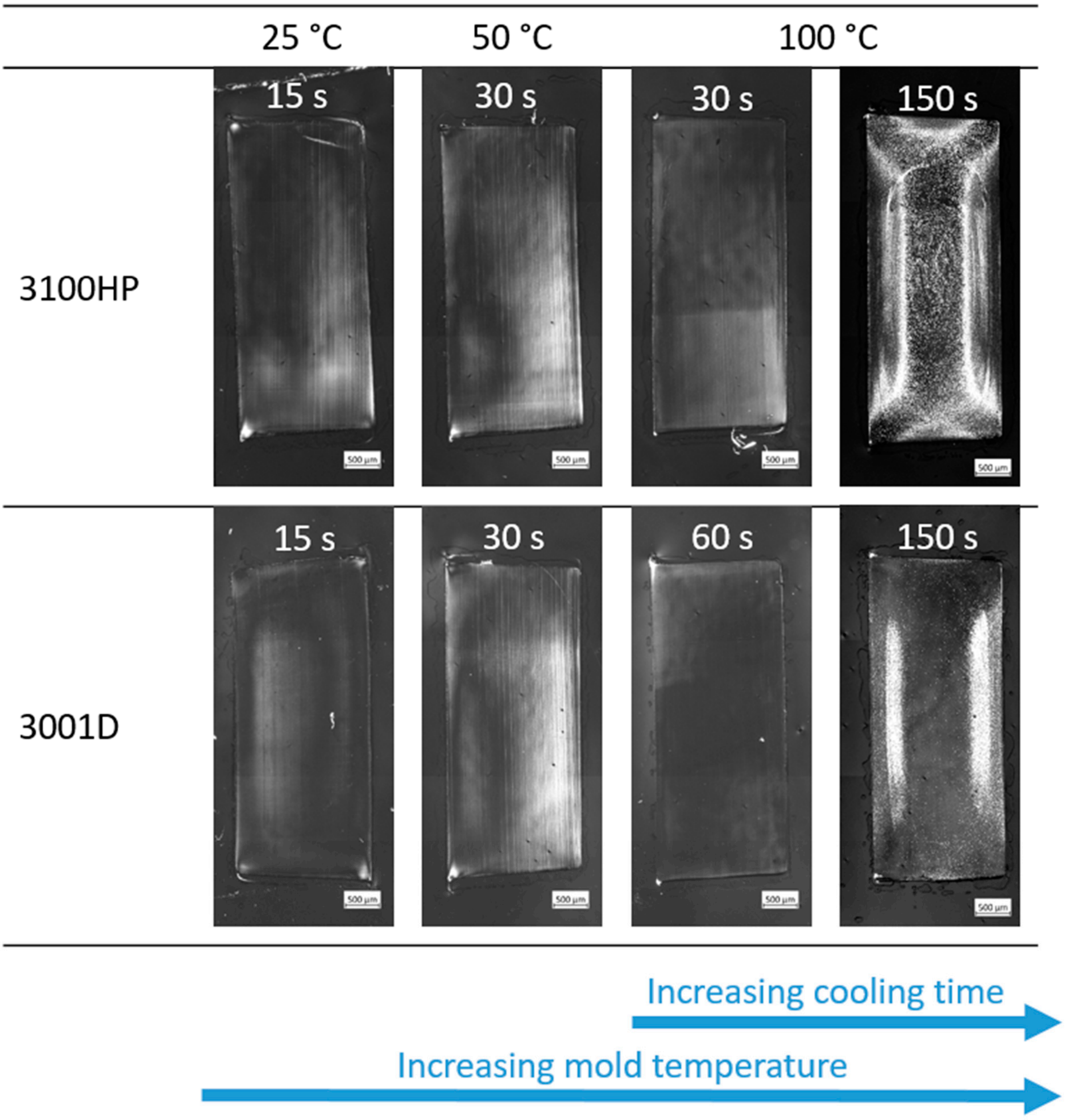

Figure S1. Polarized light microscope images of sample cross sections of PLA grades 3100HP (top row) and 3001D (bottom row) processed by injection molding with different mold temperatures and different cooling times of 15 s, 30 s, and 150 s but constant melt temperature of 210 °C.

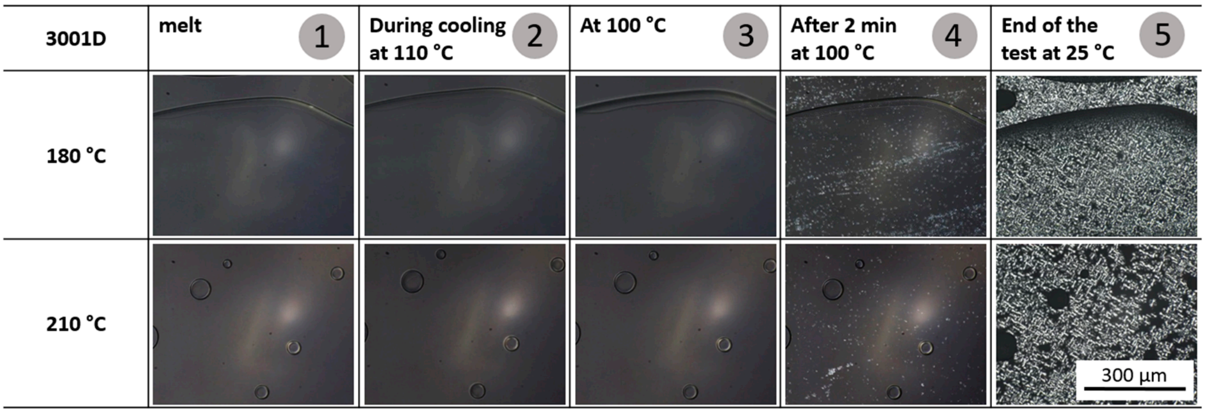

Figure S2. Light microscopic images of the material 3001D during the hot stage experiments in the molten state, during cooling to 100 °C, at the beginning of isothermal crystallization at 100 °C, after 2 min at 100 °C and at the end of the test at room temperature starting from a 180 °C (top row) and 210 °C (bottom row) hot melt.

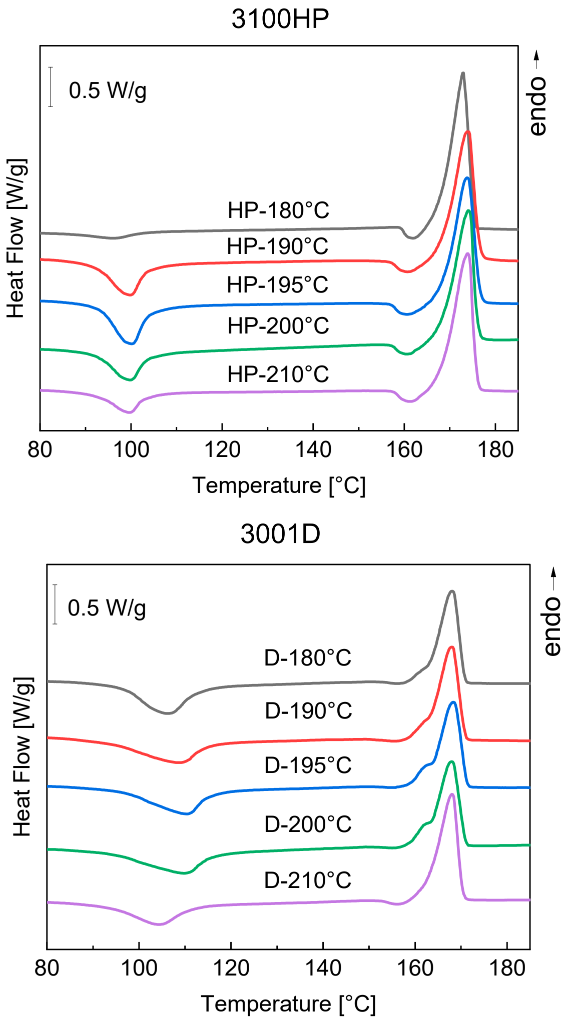

Figure S3. DSC curves of the first heating cycle of injection molded tensile bars made of PLA 3100HP and PLA 3001D produced with melt temperatures of 180 °C, 190 °C, 195 °C, 200 °C, and 210 °C and cooled in a temperature-controlled mold at 100 °C for 150 s.
